# Patients’ experiences of consultations with physician associates in primary care in England: A qualitative study

**DOI:** 10.1111/hex.12542

**Published:** 2017-04-21

**Authors:** Mary Halter, Vari M. Drennan, Louise M. Joly, Jonathan Gabe, Heather Gage, Simon de Lusignan

**Affiliations:** ^1^ Faculty of Health Social Care and Education Kingston University and St George's University of London London UK; ^2^ Social Care Workforce Research Unit King's College London UK; ^3^ Centre for Criminology & Sociology School of Law Royal Holloway University of London Egham UK; ^4^ School of Economics University of Surrey Guildford UK; ^5^ Department of Clinical and Experimental Medicine University of Surrey Guildford UK

**Keywords:** General Practice, Patient Acceptance of Health Care,, Patient Satisfaction, Physician Assistants, physician associates, Primary Health Care

## Abstract

**Background:**

Physician associates are new to English general practice and set to expand in numbers.

**Objective:**

To investigate the patients’ perspective on consulting with physician associates in general practice.

**Design:**

A qualitative study, using semi‐structured interviews, with thematic analysis.

**Setting and participants:**

Thirty volunteer patients of 430 who had consulted physician associates for a same‐day appointment and had returned a satisfaction survey, in six general practices employing physician associates in England.

**Findings:**

Some participants only consulted once with a physician associate and others more frequently. The conditions consulted for ranged from minor illnesses to those requiring immediate hospital admission. Understanding the role of the physician associate varied from ‘certain and correct’ to ‘uncertain’, to ‘certain and incorrect’, where the patient believed the physician associate to be a doctor. Most, but not all, reported positive experiences and outcomes of their consultation, with some choosing to consult the physician. Those with negative experiences described problems when the limits of the role were reached, requiring additional GP consultations or prescription delay. Trust and confidence in the physician associate was derived from trust in the NHS, the general practice and the individual physician associate. Willingness to consult a physician associate was contingent on the patient's assessment of the severity or complexity of the problem and the desire for provider continuity.

**Conclusion:**

Patients saw physician associates as an appropriate general practitioner substitute. Patients’ experience could inform delivery redesign.

## 1 BACKGROUND

Health care is labour‐intensive, and in the face of medical shortages and financial constraints, health‐care systems have designed new roles within health‐care teams to ensure delivery of care.^1^ One category of these is the mid‐level practitioner^2^ positioned to provide clinical services which may be a substitute for, delegated from or an enhancement of medical practitioner services.^3^ One such mid‐level practitioner is the physician associate (PA), previously known as physician assistant, in England and the wider United Kingdom (UK).^4^ The physician assistant role developed in the United States of America (USA) in the 1960s with over 86,000 PAs employed in all health‐care settings, including primary care, in 2015.^5^ PAs are trained in the medical model to diagnose, treat and refer autonomously, as agreed with their supervising physician, in line with local legislation.^5^ Building on the model from the USA, PAs have been introduced to other health‐care systems such as Canada, Australia, the Netherlands, Germany and India.^6^ In the UK, the first PAs employed in the mid‐2000s in the National Health Service (NHS) were American‐trained.^7^, ^8^ The first UK‐trained PAs graduated from post‐graduate diploma courses in 2009.^4^ Unlike PAs in the USA and the Netherlands, those in the UK do not currently have the legal authority to prescribe and do not currently come within a state regulatory framework for health professionals.^9^ Concern about current and predicted shortages in the general practitioner (known in some countries as family physician) workforce, together with a policy emphasis of greater delivery of care outside of hospital, has led to recommendations for more PAs to be employed in primary care^10^ and a policy statement by the Minister of Health in England that 1,000 PAs will be employed in general practice by 2020.^11^

PAs are a recent innovation in UK general practice settings, and they have been mainly deployed to provide consultations to patients requesting urgent or same day appointments.^12^, ^13^ PAs in this setting are formally defined as dependent practitioners to the general practitioner, but can work independently in the practice health‐care team, seeing and referring patients on and reviewing clinical test results.^4^ A review of evidence regarding PAs in primary care from 1950 to 2010 found only six published studies from the United States which sought the views of patients who had consulted PAs.^14^ Of these, five studies used surveys and reported high levels of satisfaction.^15^, ^16^, ^17^, ^18^, ^19^ Within the UK, two short‐term pilot schemes to introduce US‐trained PAs to different types of services, including primary care in the NHS in England and Scotland, also reported high levels of patient satisfaction.^7^, ^8^ An observational study in England comparing PA and GP consultation records (n=932 and n=1,154, respectively), with a linked patient satisfaction survey (n=490 and n=590, respectively), conducted by the authors, also found that the majority of respondents were satisfied or very satisfied with their consultation with both PAs and GPs, and all but a very small number reported confidence and trust in the PA or GP. Eleven patients (4.1%) reported they would prefer to see a GP in future.^20^

The conceptual issues and limitations of patient satisfaction surveys are well documented.^21^, ^22^, ^23^ Satisfaction is a relative concept, based on evaluative judgements,^23^ and in the instance of such a role innovation as PAs substituting for GPs, it requires more in‐depth understanding of the dimensions upon which the judgements are being made.^23^ Calnan suggested that a conceptual framework for lay evaluation of health care should include elements of the level of experience of health care and the goals of those seeking such care.^24^ In‐depth information about patient experience can be captured using interviews.^25^ (p9). However, only one study which sought the views of patients who had consulted PAs, conducted in the USA, used interview techniques.^26^ This study reported mixed responses from patients in an area where the PA had been the sole primary care provider for the previous two years, with the patients suggesting that they would sometimes prefer to see a doctor due to a) not having confidence in the PA (not being a doctor), b) already having a doctor or c) having a long‐term condition requiring specialist care.^26^

Against this background, our study addresses the evidence gap regarding the patients’ perspective on the innovation of PAs providing general practice services, in a country where nurses are an established part of the state funded, general practice team.^27^ The study draws on the interpretative tradition^28^ and builds on our patient survey respondents’ evaluative judgements to address questions of how patients understood the role of PAs and their experience of health care provided by a PA as a mid‐level health practitioner.

## 2 METHOD

The data reported here are from a larger study which involved six general practices employing PAs across southern England and six matched practices which did not.^29^ The practices were purposively sampled to represent the different types of practice found in the UK by list size and number of practice partners, in urban and rural settings with varying levels of deprivation.^29^ Five of the practices employed only one PA, the sixth employed two; four PAs were female and three male; four had trained in the USA and three in England.

Adult patients (n=430) were given a patient satisfaction survey, which included a request to volunteer for an interview, by reception staff as they left a same day or urgent consultation with a PA. Completed volunteer forms, with contact details, were returned to the researchers. A topic guide was developed to explore issues not captured by the patient survey, that is patient choice about whether they saw a PA or not and their level of satisfaction with that experience and associated reasons; the patient's understanding about the PA role, exploring information provision and experience of seeing PAs; their experience of the PA consultation compared with their expectations of consulting a GP, probing issues of confidence and trust; how issues such as making a referral and prescribing were handled by the PA and the impact of this on the patient's experience; and their perspectives on consulting a PA and/or GP in the future.

One hundred and fifty‐two patients expressed an interest in volunteering for an interview as part of the qualitative study we report here. Of these, contact details for 43 were incomplete, 40 did not respond to the researchers’ contact attempts, and four contact details were received after recruitment had closed. Researchers made contact with 40 patients and, of these, 34 participated in an interview (all but one by telephone). Interviews lasted between 10 and 20 minutes. Four interviews were not used when it became apparent that the consultation being discussed had not been with a PA or the adult participant described a consultation for a child. With consent, the interviews were digitally recorded and transcribed. Interpretive analysis was conducted using thematic analysis^30^ by two authors (LJ and MH) with another researcher. Transcripts were read and re‐read; initial codes were developed through discussion and applied initially to a small number of transcripts, enabling further discussion and iteration of the thematic index. Coding against the index was undertaken by the same three researchers, with at least two carrying out parallel coding of each transcript. Any disagreement was addressed through discussion and further iteration to the analytical process if necessary. QSR International's NVivo 10 Software was utilized in the analytic process.

The study was approved by a UK NHS Research Ethics Committee.

## 3 FINDINGS

### 3.1 Description of participants

The thirty participants were unevenly spread across the practices (minimum two, maximum 11 per practice) but were diverse in terms of gender (12 female and 18 male), age (range from 27 to 90 years), ethnicity (nine people were of black and minority origin and the remainder were white) and socio‐economic background as defined by the Index of Multiple Deprivation for their general practice^31^ (see Table 1).

**Table 1:** Index of Multiple Deprivation of the practices at which interviewees were registered

Practice employing a PA	Number of interviewees	Decile^a^ of lower super output area^31^
1	5	Eighth
5	11	Fifth
6	3	Fourth
10	2	Second
11	6	Fifth
12	3	Second

a where the first decile is the most deprived.

One participant was a carer who had accompanied their relative to the PA consultation. The types of health condition described by the participants in consulting a PA ranged from simple conditions such as an ear canal impacted with wax, to acute illnesses requiring immediate hospital admission via an emergency department and serious conditions requiring on‐going care, such as leukaemia. The participants varied in their familiarity with the PA in their practice with 11 having consulted only once while the remaining 19 had consulted the PA previously. Of the latter, three had consulted very frequently with the PA in the management of an on‐going condition.

### 3.2 Thematic analysis

Four interlinking themes were identified as follows:

1. Variation in understanding of the role of PAs
2. Trust and confidence in the PA consultation
3. Comparison with a GP consultation
4. Patient willingness to see a PA again.

Each of these themes is described and exemplified below with quotes from the transcripts.

### 3.3 Variation in understanding of the role of physician associates

The participants described the PA role in ways that varied widely. We grouped participants’ understandings into three categories: “certain and accurate,” “certain and inaccurate” and “uncertain.” The first two groups expressed their understanding of who they had seen with clarity, although their understanding may not have been accurate.

The first group was certain they understood the role of the PA and expressed this understanding accurately in terms of it being a close relationship to doctors, but correctly realizing that it was a different role, one which meant they had a recognized education but could not do everything a doctor could do. For example:

My understanding would be somebody who's less qualified than a doctor but is able to deal with the sort of more routine things like earache I guess would be a good example of it. (Participant 15)

Participants such as these recounted well‐developed strategies within the practice of informing patients about the PA role, for example leaflets at reception and information given by the PA as soon as they entered the consulting room:

They're just like doctors. I mean when I first started to go and I saw one, a long time ago,….I asked them [the reception staff] about Physician Assistants and they gave me a leaflet and then I had a chat with the person himself, the Physician Assistant and he explained about his education and his background in America and you know I realised then that they're almost doctors, they just can't quite do everything here that they can, that a doctor could do. (Participant 17**)**

Several participants in this group appeared to describe an interpretative process whereby they had picked up on clues that the PA was not a doctor, most obviously with the issue of prescription signing having to be made by a doctor and not the PA, leading to comments such as:

I worked that [the PA not being a doctor] out myself. (Participant 29)

The second of these groups was also confident in their perception of the role of the PA, but was inaccurate. They framed their description of the PA as being closely related to a doctor, for example understanding the PA as someone in training, “almost an apprentice” (Participant 17), or as a qualified doctor from another country who is simply unable to prescribe:

Basically, as I understand it, they're basically a trained physician or trained doctor, but there's just a few things that they can't carry out, like signing the prescriptions and things like that, yeah. (Participant 28)

These participants were therefore clear that there were differences between PAs and the doctors who were their GPs, and were aware of potential reasons for these but were not aware that the PA role was not in fact that of a doctor.

The third group was uncertain about the PA role. Of concern were those who had felt confident that they had seen a GP at the time of the consultation but had learned that they had seen a PA as a result of the research process. Others in this category had understood at the time that they had seen someone referred to as a PA but had not known what that meant. There were mixed views as to whether this lack of clarity was appropriate for patients. One participant considered it to be “the right way to go about it” (Participant 19) to avoid patients having concerns about not seeing a doctor, while others expressed puzzlement and a little disquiet about not understanding at the time they had seen a PA rather than a doctor, with a sense of having been misled, as in this exemplar:

I would have liked the receptionist to be a little bit more upfront with me at the beginning when I booked the appointment, and I perhaps would have liked when I went into the room the physician assistant to actually explain the role. I don't think it would have made any difference, I still would have gone in, and I still would have, I still would have felt that the treatment of me was very good, but I feel, I feel that I would have understood a little bit more about what was happening during my treatment. I don't know why they didn't tell me, I'm not sure whether they didn't want me to think [the PA's name] wasn't a doctor and to think that [the PA's name] wasn't going to do such a good job. (Participant 03)

Analysis of the participants’ accounts therefore indicated that variability in understanding of the PA role was linked to the provision of information by the practice staff and by the PA, as well as to whether this was the first time they had seen the PA or had an on‐going relationship with them.

The analysis of this theme then leads to the interlinked issue of trust and confidence in the physician associate and the general practice in which they were located.

### 3.4 Trust and confidence in the physician associate

Participants were generally positive about trust and confidence in the physician associate and the consultation although some were more cautious or contingent. Trust and confidence appeared to be both influences on and influenced by the PA consultation through an interplay of health system (that is the NHS), their general practice and individual consultation level factors.

It was evident that confidence and trust were conferred on the PA consultation, initially, through participant's trust and confidence in the wider system of the NHS and in their own general practice, in particular its senior partners. Participants reported that they trusted their GPs to employ appropriate and competent staff and made general statements such as having confidence “in all our GPs….down there” (Participant 20). Trust was also described as engendered through knowledge of the immediacy of access to a GP by the PA in any consultation, as in this example:

I knew the difference [between the PA and the GP ] and that the help was next door [the GP] if he needed it, so I was more than happy with seeing [PA's name] and that would make me confident to see [PA's name] again. (Participant 34)

Trust also appeared to be built through the experience of positive consultations, that is, trust in the individual PA. Participants described PAs as having good consultation communication skills, having time to listen and responding appropriately, as below:

To get someone like [PA's name]; because we're in our 60s that's how doctors, doctors used to be, they knew their patients. I know they're overworked now or got too many patients but [PA's name] has this ability and I think it's a given, I think some people have it and some people don't. (Participant 14)

Participants also reported trust being built through judging the PA as competent in the clinical activities of assessing, making referrals, initiating treatments (through prescriptions for medication taken to the doctor to sign) and advising on self‐management. As in the quotation, participants were often experienced in their own health conditions and used this as the basis for their judgements:

Well they've [the PAs] never given a diagnosis that I didn't think was a good diagnosis, they've always given the right medicine in my opinion, it's always worked. So I've never, ever had a problem, that's why I feel confident with them. It's as if you're seeing a doctor. (Participant 17)

Clinical competence was also noted in the identification of additional health problems that the patient had not been aware of, as in this exemplar:

She pointed something out my dad wasn't aware of. He went with a certain complaint and then when she was examining his body she saw like a sort of a lump in his neck and she was saying, ‘Mr X, what's this?’ And he was saying, ‘Oh, no, this is because of old age,’ and she was saying, ‘I don't think so, I think I need to refer you because maybe this is linked to what you're complaining about’. (Participant 02)

Judgements about competence also appeared to be contingent on the patient's previous experience of the PA. Some participants recounted trust in seeing the PA being based on the PA having known when the presenting condition(s) required the advice or additional assessment by a GP as described here:

I had no hesitation in going to an appointment with him because I'd seen him before, so I was quite happy that he was confident and knew where his boundaries laid. (Participant 34**)**

Despite a high level of trust being expressed by many participants, this was not universal and was certainly not the immediate response of everyone beginning a consultation with a PA. Some participants expressed less trust or confidence in the PA, initially as an unknown type of professional but also subsequent to negative experiences in consultation style or outcome. Such experiences raised the issue of boundaries to professional practice and how these can have a negative impact on the participants’ experience in terms of incomplete or delayed care:

I went in there and I really was nearly in tears with the pain. He (PA) listened to me in fairness, went out of the room because he has to then run it by a doctor. I waited 20 minutes and it came back and his words to me were ‘she said you'll have to come back tomorrow’. And I had to walk out of that surgery in agony. Now that isn't satisfactory…. (Participant 14)

Analysis of the issue of trust and confidence therefore highlights mixed, sometimes conflicting, experiences, apparently influenced by prior as well as “on the day” experience.

### 3.5 Comparisons with a GP consultation

Participants were not specifically asked to compare their consultation with a PA to that of a GP but many did so in explaining their experiences in terms of what they usually received at their practice. Most participants perceived that their consultation with a PA was either no different from or was very similar to a consultation with a GP. They described being asked the same questions and given the same types of examination and investigations, as they considered they would have received from a doctor:

….I had no idea that he wasn't a fully qualified GP…..the questions he asked, he did an examination, the examination that he did for me was all really professional and exactly as I would expect him to do which is why, when I walked out of the door I said ‘thank you Doctor’ because for me he did everything I was expecting…… (Participant 03)

A notable difference was when medication needed to be prescribed. The participants had experienced different methods to organize this. One reported approach was for the PA to leave the consulting room to discuss the case with a GP and then return to the patient with the signed prescription while they waited either in the consulting room or waiting room. Participants also reported collecting the signed prescription from reception or having it faxed to the local pharmacy. The need for prescriptions to be verified and signed by a GP was reported by most participants to cause no apparent or significant delay. A small number of participants reported delays of five to ten minutes with a minority reporting longer waiting times ranging from 15 to 30 minutes. While some considered this reasonable, others felt it unacceptable as in this example:

It's quite annoying, actually, because, I mean, I feel that if people can prescribe it they should be able to sign it. (Participant 23)

### 3.6 Willingness to see a physician assistant again

The majority of participants reported that they were not offered a choice of whether they saw a PA or GP when they booked their same day/urgent appointment. For the small number of participants who described having actively sought an appointment with a PA, the reasons included a shorter waiting time to see a PA, dissatisfaction with prior appointments with GPs and trust in the PA based on previous contact.

Many participants expressed their willingness to see a PA in future consultations for any condition, while others expressed a willingness to return to consult a PA as conditional on the problem. Minor conditions or less trivial complaints, for example, were seen as appropriate for a PA consultation. Participants who reported more complex conditions or medication requirements felt this was something for which they would need to consult a doctor, as illustrated here:

I think if it was just a general complaint he [a cared for relative] wouldn't mind seeing her [the PA] but regarding his prescription, he's a bit fussy about his medicine, he would prefer to see a doctor. (Participant 02)

Willingness to return to see a PA again was also influenced by participants’ motivation to help offset the pressures faced by general practitioners, as in this example:

I understand the need sometimes to take the pressure off the doctors…..so I am very aware that I don't want to take up appointments when it isn't really that necessary. So the thought that there is a role within the surgery where I could go and see somebody who isn't as pressurised as the doctor,…..is a really good thing to have in the surgery and I feel that I would be happy to utilise that again, definitely. (Participant 03**)**

For some participants, regardless of how satisfied they were with the PA consultation, maintaining continuity of care with a particular professional was equally if not more important than having a preferred type of practitioner. Consequently, if a participant had already consulted a GP about a particular problem, their preference was to consult them again. There were examples, however, where participants were choosing the PA to provide that continuity of care, giving positive accounts of the PA's ability to recall details such as a medical and family history, as well as the PA being seen as part of the community. For example:

I'm trying to make her [the PA] my regular, I say doctor, but my regular person I see at the surgery…she seems to understand my needs. I get on really well with her……… As I said, because it's a [type of practice], you're sort of shuttled around from doctor to doctor. You don't really get to make a relationship with anybody, and appointments are very quick as well, as in, you're sort of shuffled off really quickly, like a conveyor belt. (Participant 21)

## 4 DISCUSSION

Our findings presented differing patient experiences of consultations with PAs, although most were presented positively. Participants in general were unworried about the GP's task being substituted by a PA who appeared to act similarly to a GP, and who inspired high trust and confidence. However, participants were displeased if the role was not explained to them, feeling deceived by their practice and the PA. Many felt that the PA was competent to perform a GP's role, but were sometimes frustrated by the restrictions around the role, particularly the inability to prescribe. Willingness to see the PA again was differentiated by presenting condition, as well as by experience and views on continuity of care.

This article has presented greater depth of understanding of the patient's perspective, as to the experience of consulting with a new type of health practitioner, a PA, who was substituting for a doctor in general practice. While the findings were broadly reflective of the larger survey's results,^29^ the qualitative findings extend the knowledge available to those interested in the development and changes in skill mixes in providing same day or urgently requested primary care consultations – traditionally provided by doctors.^32^

**Figure 1:**
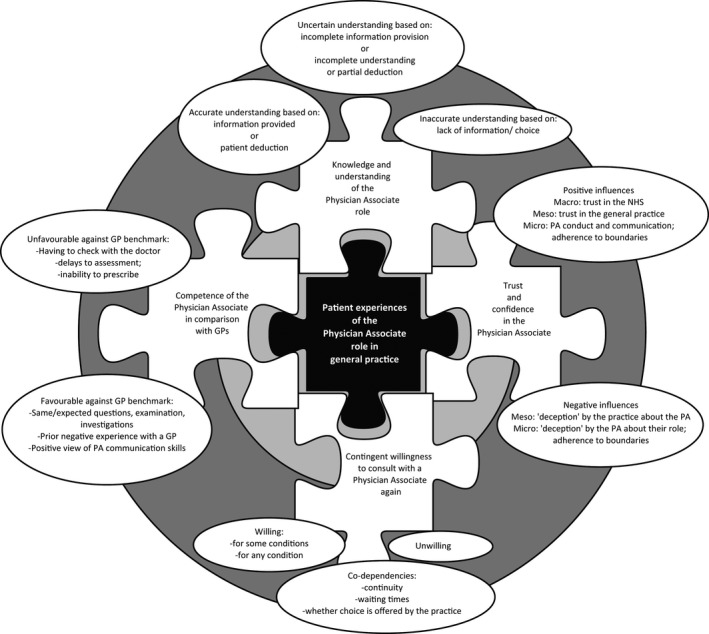
A representation of the interlinking influences on and impacts of patients’ experiences of a physician associate in general practice in England

Interlinking influences on and impacts of patients’ experiences have been identified which we present as a theoretical model illustrated in Figure 1 and discussed below.

The diversity of patients’ understandings of the professional role of the PA ranging from “certain and accurate,” through “uncertain” to “certain and inaccurate,” has been identified in other UK studies of substitution by nurse practitioners for GPs^33^, ^34^ and also in primary care dental services in the substitution of dentists by dental therapists.^35^ It was evident from the participants’ accounts that the different forms of information used by general practices to explain the role had only been partially successful in ensuring that patients understood the nature of the physician associate substituting for the doctor. The absence of prior warning and explanation created situations in which confidence in the clinical care from the PA and in the general practice as a whole was at risk. Confidence and trust are linked concepts.^36^ In health care characterized “by uncertainty and an element of risk regarding the competence and intentions of the practitioner on whom the patient in reliant”^37^ (p2), trust is considered to be crucial. It was evident that where patient confidence in the PA, was apparent, it derived from the public health system, noted in one other substitution study,^35^ but primarily from the general practice itself, as well as from the actions of the PAs themselves. Development of trust in nurse substitutes for doctors, through actual consultations, has been noted before.^33^, ^38^ We see a close relation to the model of Rowe and Calnan,^39^ who not only describe the interplay of different levels of trust, but also consider that trust relations in the NHS are increasingly based not only upon traditional clinician–patient roles of embodied, affective trust arising from status‐based reputation, relationships and interaction, but also upon informed, cognitive trust arising from rational judgements and performance, that is, trust is conditional.^39^

On a more practice‐based level, while the majority of participants confirmed a positive view of the consultations with PAs, there were those reporting less positive experiences. These were consultations in which the boundaries of the PA's knowledge or jurisdiction had been reached, resulting in a transfer of the patient to the doctor or unacceptable delays in obtaining signed prescriptions. Similar patient views have been expressed regarding nurse practitioners substituting for GPs, which has resulted in repeat consultations and more time being spent by patients in more visits to the general practice.^33^, ^38^ In the UK, a parliamentary Health Select Committee report has recommended that physician associates should be included in state regulatory processes as a matter of urgency,^40^ with the objective particularly of allowing the issue of prescribing rights to be addressed. While this would address some of the concerns our participants raised, it would not eliminate all experiences of episodes of care not being able to be completed at one visit.

Chapple et al^38^ suggested that the way in which patients accepted seeing a nurse rather than a doctor was in having their needs met in a way they expected a GP would have carried out. Similarly, in our study, it was evident that patients constructed the new role of the PA in the context of their understanding of the medical role and their willingness to consult the PAs in the future was contingent on their own view of who was needed to treat their presenting problem, alongside a desire for receiving the continuity of care that they considered used to be provided by their GP. There are different elements in continuity of care – relational as well as the management of the health condition.^41^ As the organization of general practice in the UK has changed, achieving continuity of care has become more difficult.^41^ Some participants offered insights that the consulting style of the PAs together with the perceived ease of access made them a preferred alternative to the GP. Ease of access, in terms of waiting time, is reported elsewhere as related to the concept of acceptance of seeing an alternative primary care provider to the physician.^42^ These authors report that, although a physician remained the first choice of provider for about half of the respondents, acceptance of seeing a PA or a nurse practitioner increased as the wait to see a physician increased in less urgent clinical care scenarios. Such support was not unanimous amongst their participants and varied by previous experience of physician assistants or nurse practitioners, and by income group, type of health insurance, age and ethnicity of the patient. Our qualitative data do not allow us to consider the role of such variables, and the issue of point of care cost to the patient is not relevant to the UK context; however, we do also report a discerning approach from patients about the choices they make – when offered a choice – to seeing physician associates or GPs for different clinical conditions. The extent to which this holds in practices which organize in different ways, for example, personal patient lists for GPs, would require exploration.

This study was limited in that the volunteer participants were self‐selecting, rather than purposively selected; however, they represented diversity in their characteristics and experiences of PA consultations. Our practices and PAs were also volunteers and were small in number; we do not claim that these findings are generalizable, but the numbers of PAs in primary care are currently small and we achieved a range of practices. We chose to use telephone methods to overcome logistical problems which added to the immediacy following the consultation. We are aware that, while there are suggestions from some studies that telephone interviews can yield lower quality in terms of missed reporting^43^, with the interviewer having no visual cues,^44^ others conclude that the same amount and quality of data can be gathered in telephone and face‐to‐face interviews.^45^, ^46^

## 5 CONCLUSION

Patients’ experiences of new health‐care professionals when substituting for another's role are important for understanding public acceptability and for embedding the new role. Largely positive views reported here of 30 PA consultations, in six GP practices in England, when seen as similar to those the patients have with GPs, are tempered by other views containing some critique of the role and how it is being communicated. These experiences raised issues around patient knowledge and understanding of the jurisdiction of new roles and highlighted a desire for continuity with a trusted clinician. Underpinning these was a gap regarding patient choice. Maintenance of trust and confidence in the general practice and the professionals in various roles employed within it require recognition and prominence in the organizational delivery of the general practice. Qualitative analyses can provide valuable insight into the effectiveness of health system transformations. Exploration of patient experience provides insights into the strengths and limitation of the PA in primary care.

## CONFLICT OF INTEREST

No conflicts of interest have been declared.
